# Long-Term Outcome of Myocardial Protection in Heart Transplantation: Comparison Among 3 Different Solutions

**DOI:** 10.1093/icvts/ivaf301

**Published:** 2025-12-14

**Authors:** Fabrizio Settepani, Aldo Cannata, Igor Belluschi, Giulia Pinuccia Pisani, Michele Giovanni Mondino, Andrea Garascia, Claudio Francesco Russo

**Affiliations:** Cardiac Surgery and Heart Transplant Unit, Cardio-Thoraco-Vascular Department, ASST Grande Ospedale Metropolitano Niguarda: Azienda Socio Sanitaria Territoriale Grande Ospedale Metropolitano Niguarda, Milan, 20162, Italy; Cardiac Surgery and Heart Transplant Unit, Cardio-Thoraco-Vascular Department, ASST Grande Ospedale Metropolitano Niguarda: Azienda Socio Sanitaria Territoriale Grande Ospedale Metropolitano Niguarda, Milan, 20162, Italy; Cardiac Surgery and Heart Transplant Unit, Cardio-Thoraco-Vascular Department, ASST Grande Ospedale Metropolitano Niguarda: Azienda Socio Sanitaria Territoriale Grande Ospedale Metropolitano Niguarda, Milan, 20162, Italy; Cardiac Surgery and Heart Transplant Unit, Cardio-Thoraco-Vascular Department, ASST Grande Ospedale Metropolitano Niguarda: Azienda Socio Sanitaria Territoriale Grande Ospedale Metropolitano Niguarda, Milan, 20162, Italy; Intensive Care Unit, Cardio-Thoraco-Vascular Department, ASST Grande Ospedale Metropolitano Niguarda: Azienda Socio Sanitaria Territoriale Grande Ospedale Metropolitano Niguarda, Milan, 20162, Italy; Cardiology 2 Unit, Cardio-Thoraco-Vascular Department, ASST Grande Ospedale Metropolitano Niguarda: Azienda Socio Sanitaria Territoriale Grande Ospedale Metropolitano Niguarda, Milan, 20162, Italy; Cardiac Surgery and Heart Transplant Unit, Cardio-Thoraco-Vascular Department, ASST Grande Ospedale Metropolitano Niguarda: Azienda Socio Sanitaria Territoriale Grande Ospedale Metropolitano Niguarda, Milan, 20162, Italy

**Keywords:** heart transplantation, myocardial protection, cardiac preservation solution, primary graft dysfunction, long-term results

## Abstract

**Objectives:**

We analysed our long-term experience with heart transplantation (Htx) utilizing 3 different cardioplegic solutions.

**Methods:**

During a 20-year period, 538 adult individuals underwent isolated Htx at our institution. Ten cases in which the Organ Care System TransMedics Inc was utilized were excluded, resulting in a final cohort of 528 individuals. Patients were stratified into 3 groups according to the donor heart cardioplegic solution: Celsior (*n* = 301; reference group), HTK-Custodiol (*n* = 88), and St Thomas (*n* = 139). Mean follow-up period was 6.2 ± 5.5 years (maximum 20 years).

**Results:**

The rate of severe primary graft dysfunction (PGD) was 10.2% in the HTK-Custodiol group, significantly higher than the reference group (4.5%; *P* < .040). Overall, in-hospital mortality was 12.9%: 13.6% in the HTK-Custodiol group and 12.9% in the St Thomas group, comparable to the reference group (*P* = .803 and *P* = .924). Survival at 1, 5, and 12 years in the Celsior and HTK-Custodiol groups was 82.6 ± 2.2% vs 85.2±3.8%, 79.4 ± 2.4% vs 82.1 ± 4.3%, and 66.8 ± 3.3% vs 62.9 ± 7.3%, respectively (*P* = .706). Survival at 1, 5, and 12 years in the St Tomas group was 81.5 ± 3.4%, 71.9 ± 4.1%, and 65.5 ± 5.2%, respectively, comparable to the reference group (*P* = .640). Post-transplant rejection rate was similar among the groups.

**Conclusions:**

The use of HTK-Custodiol solution was associated with a significantly higher incidence of PGD when compared to Celsior solution, although this data had no impact on in-hospital mortality. Long-term survival and post-transplant rejection were comparable among the 3 groups. HTK-Custodiol solution should be used with caution for preservation of donor hearts.

**ERB Approval Number:**

215-29042020; May 5, 2020.

## Graphical abstract

**Figure:**
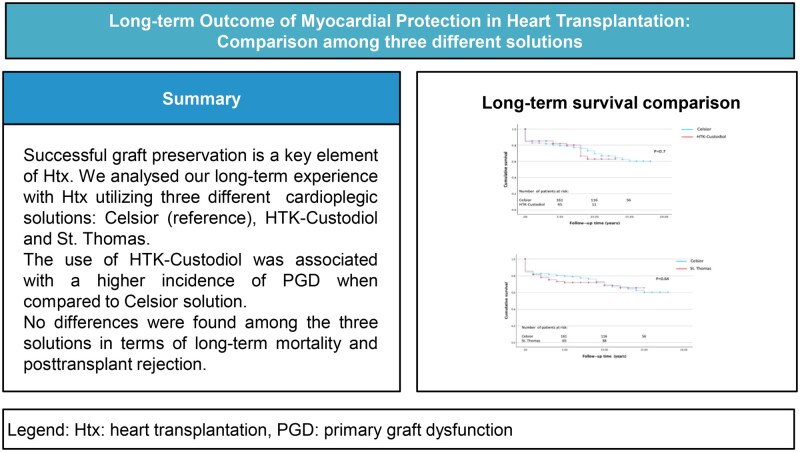

## INTRODUCTION

Heart transplantation (HTx) has become the preferred option for select end-stage heart failure patients.^1^ Effective graft preservation, typically achieved through diastolic arrest and static cold ischaemia with a 4-6 h limit, is crucial.^2^ Despite various preservation solutions, an optimal standard remains elusive.^3^ This article reports our single-center, 20-year experience with 3 different cardiac preservation solutions (Celsior, HTK-Custodiol and St Thomas) in HTx.

## METHODS

### Study design and patient population

This was an observational study, utilizing a prospectively maintained institutional database of all consecutive Htx at the Niguarda Hospital, Milan. The study was approved by the Institutional Ethic Board at Niguarda Hospital Milan (ERB approval number: 215-29042020; May 5, 2020) and individual consent was waived. The collection and use of identifiable data and biological materials prioritize individual rights, informed consent, privacy, and robust governance in accordance with the The WMA Declaration of Taipe. The ethics committee has approved the establishment and monitored ongoing use of such databases and biobanks.

From January 2002 to December 2022, 538 adult patients were consecutively submitted to isolated Htx at our institution. This period was selected because the operative technique and immunosuppressive treatment were comparable in the entire cohort. To minimize the effect of variables that could interfere with the results, 10 cases in which the Organ Care System TransMedics Inc was utilized were excluded from the study, resulting in a final cohort of 528 individuals. Patients were stratified into 3 groups according to the donor heart cardioplegic solution: Celsior (*n* = 301; reference group), HTK-Custodiol (*n* = 88), and St Thomas (*n* = 139). The perioperative variables and postoperative outcomes were retrospectively reviewed. The primary end-points were recipient severe PGD, survival, and post-transplant rejection.

### Operative technique

Grafts were harvested from beating-heart brain-dead donors. Our clinical protocols for preservation solutions were previously reported in details.^4^ Briefly, for donor heart preservation, we adopted 3 types of solutions: extracellular Celsior (Genzyme Corp., Boston, MA, United States), intracellular HTK-Bretschneider (Custodiol, Dr Franz Cohler Chemie GMBT, Bensheim, Germany), and extracellular St Thomas No. 2 (Plegisol, Hospira, Inc, United States). The choice of the solution was left to the surgeon’s discretion. Upon implantation, antegrade cold (4°C) 4:1 blood cardioplegia was administered every 20 min to hearts preserved by means of Celsior and St Thomas solutions. In accordance with the Custodiol cardioplegia administration protocol, that enables up to 3 h of safe cardioplegic arrest, no further cardioplegia was administered during implantation to hearts preserved by means this solution, unless total ischaemic time exceeded 180 min: in that case, half dose of Custodiol was re-administered.^3^^,^^5^^,^^6^ Orthotopic transplantation was invariably performed with standard left atrial cuff technique. Our approach to the primary graft dysfunction (PGD) has been previously described.^7^ Briefly: a pulmonary artery catheter was inserted after the transplantation. If this was not possible, PGD was diagnosed using echocardiographic parameters as per Kobashigawa et al.^8^ Accordingly, severe PGD was diagnosed as dependence on left or biventricular mechanical support, after excluding a discernible cause such as hyper-acute rejection, pulmonary hypertension, or known surgical complications (eg, uncontrolled bleeding). With regards to haemodynamic parameters, severe haemodynamic compromise lasting >1 hour manifested as hypotension (systolic blood pressure <90 mmHg), low cardiac output (cardiac index <2.0 L/min/m^2^), high filling pressures (right atrial pressure (RAP) >15 mmHg and pulmonary capillary wedge pressure >20 mmH), requiring ≥2 intravenous inotropic/vasopressor drugs, including high-dose norepinephrine (>0.7 μg/kg/min) or high-dose epinephrine (>0.07 μg/kg/min), indicate PGD. To support the diagnosis, an echocardiographic assessment of the left and right ventricle must be performed: in details, a low left ventricle ejection fraction (40% or less), a reduction of the tricuspid annular plane systolic excursion (13 mm or less) and RV free wall strain (−14% or less), are key indicators of PGD.

### Statistical analysis

Baseline clinical and procedural characteristics as well as clinical outcomes were compared between the groups. Continuous variables were expressed as the mean ± SD and were analysed by using the Welch *t*-test. Categorical variables were presented as percentage and were analysed with the *X*^2^ test or Fischer exact test when appropriate. Estimates for long-term survival were made by the Kaplan-Meier method. Differences between survival curves were evaluated with the log-rank statistic. Cox proportional hazard model was used to analyse the relationship between survival probability and treatment. Univariate and multivariable analysis of risk factors for late mortality was performed. For the univariate analysis several pre-operative and intraoperative variables were considered. Variables that achieved a *P*-value less than .2 in the univariate analysis were examined by using Cox proportional hazard regression. Statistical analysis was performed using SPSS version 29 (SPSS, Inc., Chicago, IL, United States).

## RESULTS

### Comparison among the groups

Pre-operative recipient and donor characteristics with statistical comparison of the 3 groups are displayed in **Tables 1 and 2**, respectively. The groups were similar for most of the variables considered. However, when compared to the reference group, patients in the St Thomas group had a significantly higher rate of pre-operative extra-corporeal membrane oxygenation (ECMO) (12.2% vs 6.4%, *P* = .040), whereas, patients in the HTK-Custodiol group had a higher rate of pre-operative ventilatory support (17% vs 8.4%, *P* = .020). With respect to the donor characteristics, no significant differences were observed among the groups for most of the variables, but smoke history, serology positive for toxoplasmosis, and cytomegalovirus were more common in the HTK-Custodiol group.

**Table 1.** Pre-Operative Recipient Characteristics

Variables	Celsior (*n* = 301) reference group	HTK-Custodiol (*N* = 88)	*P*-value	St. Thomas (*n* = 139)	*P*-value
Age (years)	49.4 (±11.2)	49.3 (±11.3)	.921	48.6 (±11.4)	.476
Gender, female	88 (29.2%)	32 (36.4%)	.203	45 (32.4%)	.505
BMI (kg/m^2^)	23.1 (±3,8)	23.6 (±3,3)	.193	23.3 (±3,4)	.518
PVRI (Woods units)	4.2 (±2,6)	4.5 (±2,4)	.323	4.1 (±2,4)	.847
Aetiology of heart failure					
Dilated cardiomyopathy	113 (37.5%)	37 (42%)	.445	52 (37.4%)	.979
Hypertrophic cardiomyopathy	16 (5.3%)	6 (6.8%)	.591	8 (5.8%)	.850
Ischaemic aetiology	93 (31%)	19 (21.6%)	.087	38 (27.3%)	.435
Valvular aetiology	14 (4.7%)	3 (3.4%)	.612	2 (1.4%)	.093
Congenital heart disease	9 (3%)	3 (3.4%)	.841	4 (2.9%)	1.0
CAV	7 (2.3%)	1 (1.1%)	.489	5 (3.6%)	.531
Restrictive cardiomyopathy	5 (1.7%)	3 (3.4%)	.309	3 (2.2%)	.712
VAD complications	9 (3%)	2 (2.3%)	.721	6 (4.3%)	.537
Emergency transplant	58 (19.3%)	19 (21.8%)	.597	33 (23.7 %)	.282
L-VAD	43 (14.5%)	8 (9.1%)	.187	26 (18.7%)	.266
ECMO	19 (6.4%)	9 (10.2%)	.228	17 (12.2%)	**.040**
Impella	4 (1.4%)	0	.578	1 (0.7%)	.564
IABP	35 (11.8%)	13 (14.8%)	.463	16 (11.5 %)	.924
Ventilator support	25 (8.4%)	15 (17%)	**.020**	12 (8.6%)	.948
Prior cardiac surgery	84 (27.9%)	21 (23.9%)	.452	41 (29.5%)	.731
Diabetes	33 (11.3%)	13 (14.8%)	.376	12 (8.7 %)	.416
Chronic renal failure	69 (23.5%)	24 (27.6%)	.432	33 (24.1%)	.882
Acute renal failure	28 (9.5%)	8 (9.2%)	.927	7 (5.1%)	.118
Dialysis	2 (0.7%)	0 (0%)	.441	2 (1.5%)	.432
CVVH	45(1.7%)	3 (3.4%)	.313	4 (2.9%)	.412
Serology					
HCV	14 (4.7%)	3 (3.4%)	.752	3 (2.2%)	.203
HIV	0	0	1.0	1 (0.7 %)	1.0
Creatinine at transplant (mg/dL)	1.21 (±0.54)	1.19 (±0.4)	.737	1.22 (±1)	.956
Bilirubin at transplant (mg/dL)	1.15 (±1.12)	1.96 (±7.33)	.322	1.83 (±6)	.209

Abbreviations: BMI = body mass index; CAV = cardiac allograft vasculopathy; CMV = cytomegalovirus, CVVH = continuous veno-venous hemofiltration; HCV = hepatitis C virus; HIV = human immunodeficiency virus; IABP = intra-aortic balloon pump; L-VAD = left ventricular assist device; PVRI = pulmonary vascular resistance index.

The values reported in bold are considered statistically significant.

**Table 2.** Donor Characteristics

Variables	Celsior (*n* = 301) reference group	HTK-Custodiol (*N* = 88)	*P*-value	St. Thomas (*n* = 139)	*P*-value
Age (years)	41.5 (±13.8)	44.2 (±12.7)	.086	43.3 (±13.1)	.190
Gender female	105 (35%)	34 (39.1%)	.482	49 (35.5%)	.911
BMI (kg/m^2^)	25 (±3.9)	25.4 (±2.1)	.446	25.4 (±3.5)	.488
Cause of death					
Head trauma	45 (15.1%)	8 (9.2%)	.163	22 (16.6%)	.763
SAH	134 (44.8%)	43 (49.4%)	.448	59 (43.4%)	.780
Polytrauma	64 (21.4%)	25 (28.7%)	.153	27 (19.9%)	.712
Comorbidities					
Hypertension	61 (22.9%)	21 (25%)	.696	23 (18.7%)	.345
Diabetes	2 (0.8%)	2 (2.4%)	.244	1 (0.8%)	1.0
Obesity (BMI >30)	5 (1.9%)	3 (3.6%)	.404	2 (1.6)	1.0
Cocaine abuse	7 (2.6%)	5 (5.9%)	.171	9 (7%)	.055
Smoke history	53 (19.9%)	27 (32.1%)	**.020**	34 (27.4%)	.098
Inotropic support					
LD	136 (47.9%)	35 (42.2%)	.358	50 (39.1%)	.109
MD	80 (28.2%)	18 (21.7%)	.240	34 (26.6%)	.736
HD	24 (8.5%)	9 (10.8%)	.503	13 (10.2%)	.575
Serology					
Toxoplasmosis	7 (2.5%)	7 (8.3%)	**.021**	8 (6.3%)	.084
CMV	17 (5.9%)	13 (15.3%)	**.005**	17 (13.3%)	**.019**
HCV	3 (1%)	2 (2.4%)	.320	1 (0.8%)	1.0
Severe hypotension/cardiac arrest	74 (27%)	29 (33.7%)	.229	37 (30.3%)	.497
Cardiac arrest time (min)	3.3 (±1.19)	2.4 (±2.1)	.411	2.1 (1.6)	.216
Positive blood culture	4 (1.6%)	4 (5.2%)	.089	3 (2.1)	.137
Blood transfusion	133 (59.1%)	49 (64.5%)	.402	65 (58.6%)	.921
ICU time (days)	3.40 (±3.6)	3 (±3.1)	.368	3.7 (±3.4)	.489
CPK total (U/L)	509 (±80)	411 (±45)	.155	430 (±62)	.395
CPK-MB (U/L)	21 (±5)	15 (±3)	.723	31 (±3)	.633
Serum Na^+^ (mEq/L)	147 (±12)	149 (±9)	.335	148 (±9)	.439
Serum K^+^(mEq/L)	4 (±2.7)	3.8 (±0.5)	.193	3.9 (±0.6)	.407
Central venous pressure (mmHg)	7 (±3)	8 (±3)	.528	8 (±3)	.335

Abbreviations: BMI = body mass index; CMV = cytomegalovirus; CPK = creatine phosphokinase; LD = low dose, MD = medium dose; HD = high dose; HCV = hepatitis C virus; ICU = intensive care unit; SAH = subarachnoid haemorrhage.

The values reported in bold are considered statistically significant.

Intraoperative data as well as postoperative outcomes for each group are summarized in **Table 3**.

**Table 3.** Intraoperative Data and Postoperative in-Hospital Outcomes

Variables	Celsior (*n* = 301) reference group	HTK-Custodiol (*N* = 88)	*P*-value	St. Thomas (*n* = 139)	*P*-value
Female donor/male recipient sex mismatch	38 (12.6%)	9 (10.2%)	.544	19 (13.7%)	.763
Donor/recipient ratio BMI	1.106 (±0.225)	1.096 (±0.23)	.620	1.11 (±0.235)	.887
Donor-recipient pHM undersize >15%	28 (9.5%)	5 (5.9%)	.303	9 (6.7%)	.344
CPB time (min)	208 (±78)	224 (±80)	.114	212 (±63)	.623
Total ischaemic time (min)	185 (±53)	180 (±50)	.459	186 (±51)	.822
ICU LOS (days)	12.3 (±3.8)	11.7 (±1.4)	.849	10.8 (±1.2)	.556
Transfusion requirements (units)					
RBC	3.9 (±0.6)	5.1 (±0.8)	.175	5 (±0.8)	.164
Platelets	0.6 (±0.1)	0.6 (±0.3)	.899	0.5 (±0.2)	.498
FFP	2.1 (±0.4)	3.1 (±0.8)	.322	2 (±0.3)	.787
ECMO	13 (4.5%)	9 (10.2%)	**.046**	5 (3.6%)	.679
IABP	41 (13.4%)	26 (29.5%)	**.001**	29 (21.2%)	.074
Primary graft dysfunction (severe)	13 (4.5%)	9 (10.2%)	**.040**	5 (3.6%)	.696
CVVH	46 (15.7%)	19 (21.6%)	.198	23 (18.6%)	.774
Chronic renal failure (new onset, including dialysis)	23 (7.8%)	3 (3.8%)	.215	12 (9.4%)	.588
Dialysis (excluding pre-operative)	5 (1.7%)	0 (0%)	.589	0 (0%)	.320
TND	22 (7.6%)	6 (6.9%)	.817	11 (8.1%)	.872
PND	7 (2.4%)	3 (3.4%)	.704	4 (2.9%)	.762
Sternal wound infection	9 (3.1%)	1 (1.1%)	.464	2 (1.5%)	.308
UTIs	5 (1.7%)	2 (2.3%)	.74	3 (2.2%)	.755
Pneumonia	48(16.6%)	15 (17.2%)	.922	25 (18.4%)	.685
Tracheostomy	15 (5.2%)	3 (3.4%)	.775	8 (5.9%)	.819
In-hospital mortality	38 (12.6%)	12 (13.6%)	.803	18 (12.9%)	.924

Abbreviations: BMI = body mass index; pHM = predicted heart mass; CPB = cardiopulmonary bypass; CVVH = continuous veno-venous hemofiltration; ECMO = extracorporeal membrane oxygenation; FFP = fresh frozen plasma; IABP = intra-aortic balloon pump; ICU = intensive care unit; LOS = length of stay; PND = permanent neurologic deficit; RBC = red blood cells; TND = transient neurologic deficit; UTIs = urinary tract infections.

The values reported in bold are considered statistically significant.

There were no significant differences among the groups regarding intraoperative variables including total ischaemic time.

With regards to postoperative outcomes, patients in the HTK-Custodiol group had a higher rate of ECMO (10.2% vs 4.5%, *P* = .046) and intra-aortic balloon pump (IABP) (29.5% vs 13.4%., *P* = .001).

### Primary graft dysfunction

Patients in the HTK-Custodiol group had a higher rate of severe primary graft dysfunction (10.2% vs 4.5%, *P* = .040). Instead, the rate of severe PGD in the St Thomas group was found to be comparable to the reference group (3.6% vs 4.5%, *P* = .696). The mean values of the main haemodynamic and echocardiographic parameters at the time of severe PGD diagnosis were: systolic blood pressure = 74 (±6) mmHg, cardiac index = 1.3 (±0.3) L/min/m^2^, pulmonary capillary wedge pressure = 28 (±7) mmHg, left ventricle ejection fraction = 22 (±5)%, RV free wall strain = −11 (±3)%.

**Table 3** displays in details intraoperative and postoperative in-hospital outcomes.

### Survival analysis

**Figure 1.**
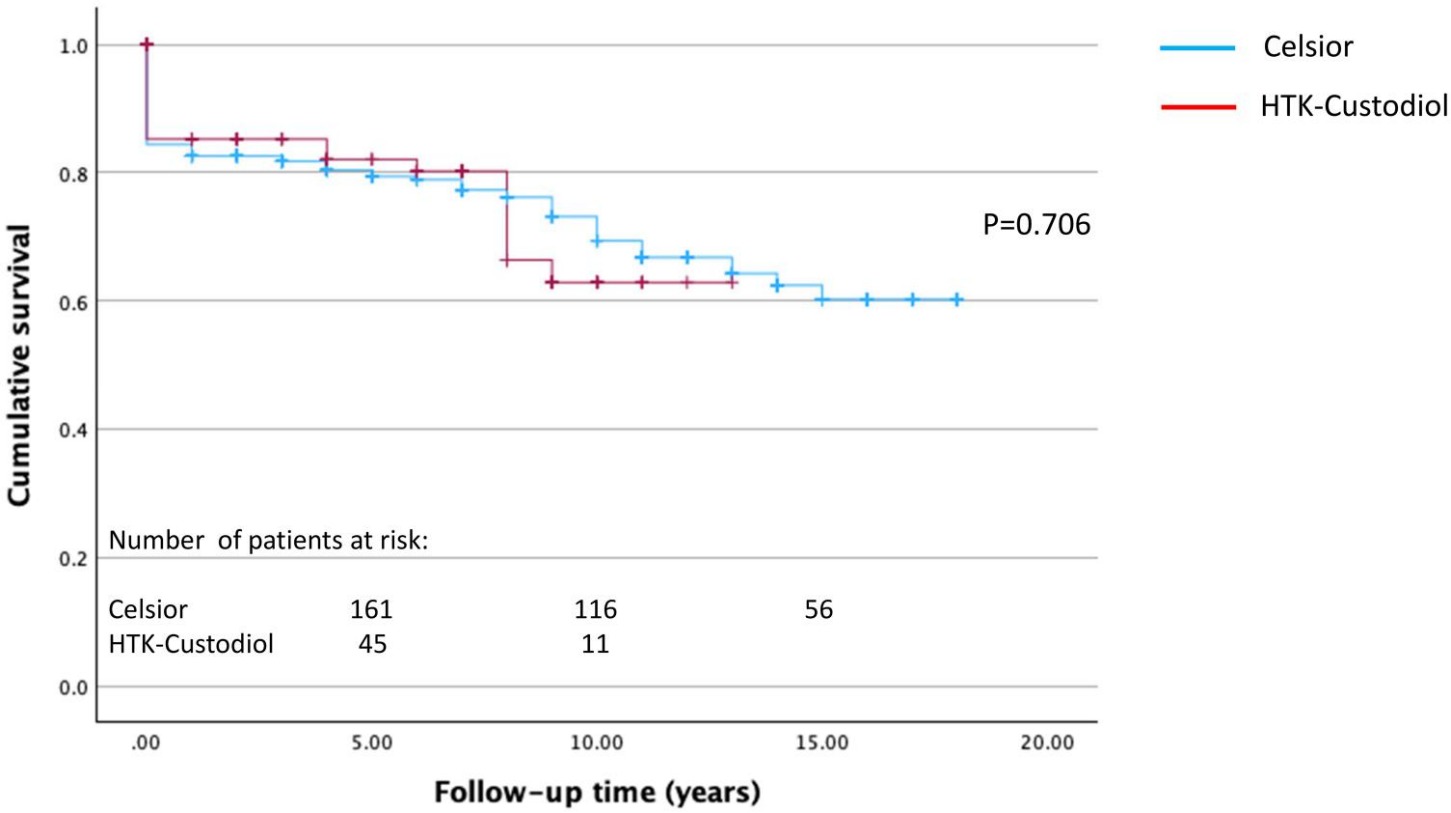
Kaplan-Meier Post-Transplant Survival Curves in Celsior and HTK-Custodiol Groups

**Figure 2.**
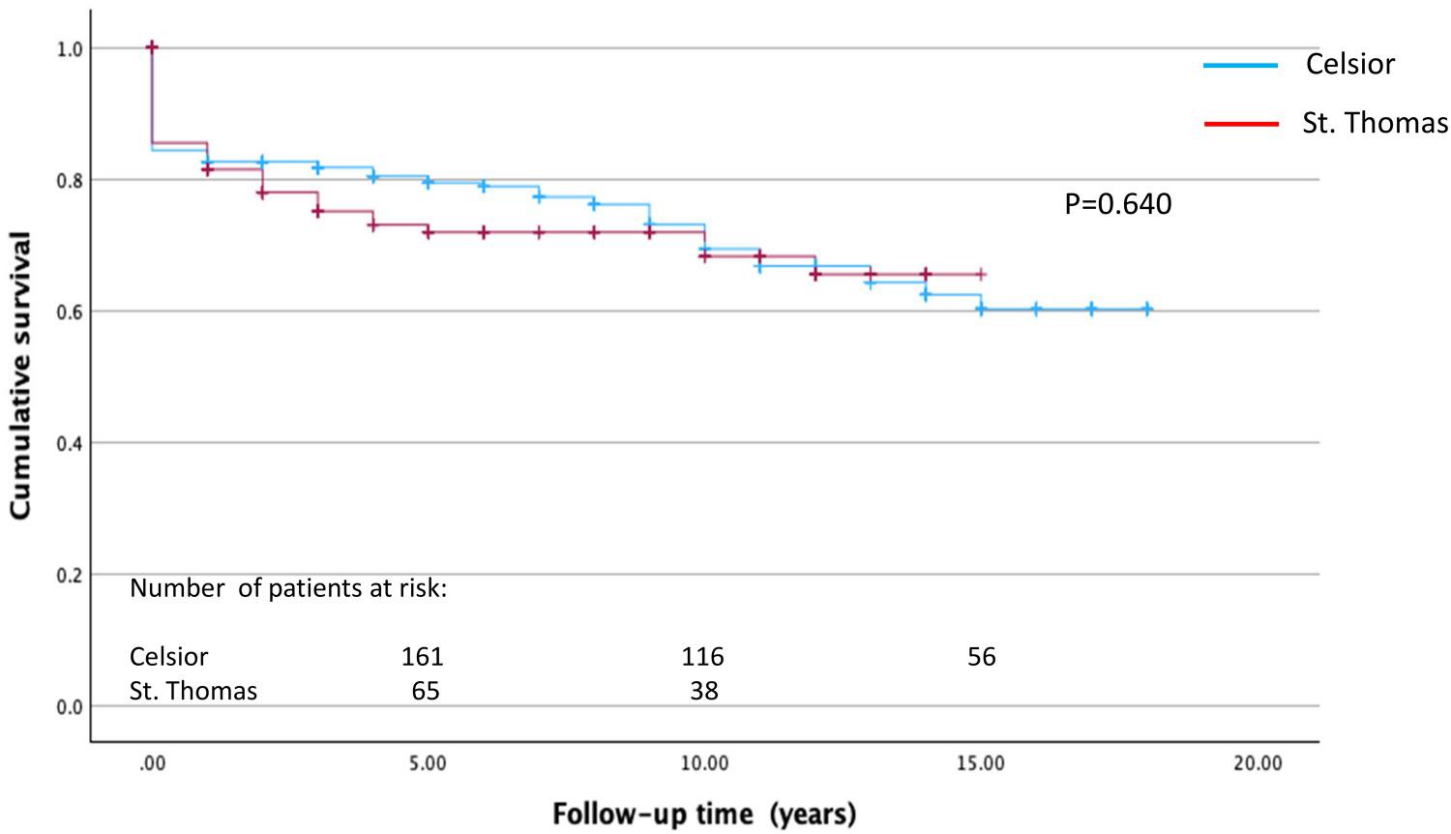
Kaplan-Meier Post-Transplant Survival Curves in Celsior and St Thomas Groups

Overall, in-hospital mortality was 12.9%; 13.6% in the in HTK-Custodiol group and 12.9% in the St Thomas group, comparable to the reference group (*P* = .803 and *P* = .924). Mean follow-up period was 6.2 ± 5.5 years (maximum follow-up time 20 years; 98% complete). Survival at 1, 5, and 12 years in the Celsior and HTK-Custodiol groups was 82.6 ± 2.2% vs 85.2 ± 3.8%, 79.4 ± 2.4% vs 82.1 ± 4.3%, and 66.8 ± 3.3% vs 62.9 ± 7.3%, respectively (*P* = .706) (**Figure 1**). Survival at 1, 5, and 12 years in the St Tomas group was 81.5 ± 3.4%, 71.9 ± 4.1%, and 65.5 ± 5.2%, respectively, comparable to the reference group (*P* = .640) (**Figure 2**).

Using the Celsior solution as the comparator for survival probability, the hazard function analysis showed the HTK-Custodiol and the St Thomas not statistically distinct from the Celsior (HR 1.06, *P* = .795 and HR 1.03, *P* = .748, respectively).

### Post-transplant rejection

Overall, the rate of post-transplant rejection was 51.5%. The rejection was cellular in the 49.7% of the cases and humoral in the 5.3%. No significant difference was detected in the HTK-Custodiol and St Thomas groups when compared to the reference group. Detailed data are shown in **Table 4**.

**Table 4.** Post-transplant Rejection

Variables	Celsior (*n* = 301) reference group	HTK-Custodiol (*N* = 88)	*P*-value	St. Thomas (*n* = 139)	*P*-value
Cellular rejection	142 (48.8%)	48 (57.8%)	.139	67 (50.4%)	.572
Humoral rejection	15 (5.2%)	3 (3.6%)	.533	9 (6.8%)	.493

### Univariate and multivariable analysis

Univariate and multivariable Cox regression model analyses were performed to identify risk factors for late mortality. In the univariate analysis, many factors have been found to be significant. Multivariate analysis identified increasing recipient and donor age, recipient chronic renal failure, total ischaemic time, and severe PGD as independent risk factors for late mortality. Details are provided in **Table 5**.

**Table 5.** Univariate and Multivariable Cox Regression Model of Risk Factors for Late Mortality

	Univariate analysis			Multivariable analysis		
	OR	95% CI	*P*-value	HR	95% CI	*P*-value
Recipient factors						
Age (years)	1.047	1.024-1.069	**<.001**	1.027	1.009-1.046	**.003**
Sex female	1.558	1.117-2.175	**.006**			
BMI (kg/m^2^)	1.011	0.957-1.069	.685			
Aetiology of heart failure						
Dilated cardiomyopathy	1.252	0.814-1.925	.306			
Hypertrophic cardiomyopathy	0.489	0.162-1.474	.195			
Ischaemic aetiology	1.594	1.018-2.496	**.041**			
Valvular aetiology	1.140	0.337-3.860	.762			
Congenital heart disease	1.140	0.337-3.860	.762			
CAV	1.140	0.337-3.860	.762			
Restrictive cardiomyopathy	0.374	0.045-3.137	.681			
VAD complications	0.168	0.022-1.299	.073			
Emergency transplant	0.455	0.256-0.810	**.006**			
Pre-operative L-VAD	1.136	0.649-1.989	.655			
Pre-operative ECMO	0.771	0.350-1.697	.517			
Pre-operative Impella	6.907	0.717-67.651	.087			
Pre-operative IABP	0.625	0.308-1.268	.190			
Pre-operative ventilator support	1.096	0.517-2.325	.811			
Prior cardiac surgery	1,243	0.787-1.963	.351			
Diabetes	1.048	0.510-2.152	.899			
Chronic renal failure	1.994	1.238-3.211	**.004**	1.518	1.059-2.177	**.023**
Acute renal failure	0.907	0.405-2.031	.813			
Dialysis	4.525	0.406-50.371	.227			
CVVH	1.116	0.274-4.538	1.0			
Donor factors						
Age (years)	1.019	1.003-1.035	**.021**	1.015	1.001-1.030	**.040**
Gender female	1.152	0.856-1.552	.342			
BMI (kg/m^2^)	1.025	0.970-1.083	.381			
Cause of death						
Head trauma	1.206	0.706-2.242	.433			
SAH	0.858	0.559-1.315	.481			
Polytrauma	0.926	0.549-1.563	.773			
Comorbidities						
Hypertension	1.008	0.594-1.711	.977			
Diabetes	1.084	0.097-12.079	1.0			
Obesity (BMI >30)	0.428	0.049-3.709	.699			
Cocaine abuse	1.985	0.702-6.612	.253			
Smoke history	0.908	0.525-1.570	.730			
Inotropic support HD	1.169	0.561-2.435	.676			
Severe hypotension/cardiac arrest	1.090	0.673-1.765	.725			
Cardiac arrest time (min)	1.008	0.987-1.029	.470			
Cardioplegic solution						
Celsior	1.102	0.704-1.727	.671			
HTK-Custodiol	1.008	0.992-1.024	.306			
St. Thomas	0.931	0.592-1.464	.758			
Transplant data						
Total ischaemic time	1.005	1.001-1.009	**.025**	1.004	1.001-1.007	**.018**
Severe PGD	3.087	1.407-6.772	**.003**	3.253	1.805-5.863	**<.001**

The values reported in bold are considered statistically significant.

## DISCUSSION

The variety of myocardial preservation solutions and strategies utilized by the heart transplant centres reflects the lack of consensus on the optimal methods.^4^^,^^9^ Preservation solutions can be grouped broadly into extracellular and intracellular. While extracellular solutions induce cardiac arrest via depolarization, the intracellular solutions reduce the membrane potential resting state, thus preventing the generation of action potentials. Celsior is an extracellular high sodium and low potassium solution, with glutamate as energy substrate and reduced glutathione and histidine as oxygen-free radical scavengers. HTK-Custodiol is classified as an intracellular, crystalloid cardioplegia due to its low sodium and calcium content. Sodium depletion of the extracellular space causes a hyperpolarization of the plasma membrane of myocytes, inducing cardiac arrest in diastole. The main characteristics of this solution is that it allows a safe cardioplegic arrest time up to 3 h.^3^^,^^5^^,^^6^^,^^10^ Hachida et al indicated that the promotion of anaerobic glycolysis during ischaemic by HTK-Custodiol cardioplegia results in prolonged preservation of the energetic and contractile function of the heart.^11^ Therefore, following the cardioplegia administration protocol uniformly recognized in the literature, we repeated a second half dose (half the initial dose) of HTK-Custodiol only if the total ischaemia time exceeded 180 min.^12^ St Thomas No. 2 is a crystalloid extracellular cardioplegia which provides a rapid diastolic cardiac arrest by high potassium and magnesium concentration as well as by the stabilizing effect of procaine hydrochloride on membrane.

HTK-Custodiol cardioplegia in donors significantly increased severe PGD incidence in our study, roughly doubling the rate compared to the reference group. Despite this, comparable in-hospital mortality rates between the groups (13.6% vs 12.6%; *P* = .803) were observed. While PGD is a clinical diagnosis of severe ventricular dysfunction within 24 h post-transplant with no identifiable secondary cause, microscopic examination can show ischaemia-reperfusion injury and early inflammatory responses. Common histological features observed in PGD include myocyte oedema, contraction band necrosis, interstitial haemorrhage, and varying degrees of inflammatory cell infiltration, predominantly neutrophils and macrophages. These findings reflect the acute cellular damage and innate immune activation that occur during the donor heart preservation and reperfusion.^13^ However, it is important to note that these histological changes are often non-specific and can overlap with other forms of graft injury, such as early rejection or donor-related myocardial abnormalities.

Regarding the recipient characteristics, although the pre-operative ECMO rate was significantly higher in the St Thomas group, this finding had no impact neither on mortality nor on PGD incidence. While the most of the literature has shown that pre-operative ECMO support is associated with higher morbidity and mortality,^14^ selected studies have demonstrated similar post-transplant survival.^15^ Although our data are not strong enough to demonstrate a protective effect of the St Thomas solution in this subgroup of patients, this finding invites reflection.

Our data did not show an improvement in mid- and long-term survival associated with 1 of the 3 solutions. In a recent comparison of HTK-Custodiol and St Thomas as cardiac preservation solution in HTx, Dulguerov et al^16^ found no difference in the rate of severe post-transplant PGD and a significantly improved mid-term survival associated with the use of HTK-Custodiol solution. However, they used the 2 solutions separately in different eras (2009-2015 St Thomas, 2016-2020 HTK-Custodiol) and this may have significantly influenced the results, considering that the mean follow-up of the HTK-Custodiol patients was considerably shorter than for St Thomas patients (3.1 vs 7.0 years). Seen from the meta-analysis of results from 147 hearts comparing HTK-Custodiol with Celsior solution, heart dysfunction and in-hospital mortality had no statistical difference in the 2 preservation solutions but the Celsior tended to have better outcomes.^17^ Karduz et al^18^ evaluated the effect of HTK-Custodiol, St Thomas, and Del Nido solutions functionally and biochemically in a rat model of donor heart. HTK-Custodiol administration led to reduced myocardial contraction, decreased ATP level, increased TNF-α, and increased troponin I levels. The authors conclude that HTK-Custodiol had the weakest protection in inflammation, oxidative stress, and myocardial contractile strength. In a similar transplantation model, an imbalance between antioxidants and oxidants following HTK-Custodiol administration has shown to be associated with oxidative stress.^3^ Although these studies provide solid data, the inherent limitations of the animal model do not allow us to transpose the results directly to humans.

With respect to post-transplant rejection, we found no significant difference among the groups. Accordingly, Carter et al,^19^ in a large recent study, failed to show significant difference in rejection when comparing Wisconsin, Celsior, and HTK-Custodiol solutions, whereas, in another comparative study among HTK-Custodiol, Celsior, and Viaspan solutions,^20^ Celsior allowed for better post-transplant heart recovery and accounted for less incidence for vasculopathy and chronic rejection in the mid-term follow-up.

In the multivariate analysis for late mortality, none of the 3 solutions used was found to be an independent risk factor. This finding suggests that, in the context of contemporary HTx practices and patient management, the choice of these specific cardioplegia solutions may not be a primary determinant of long-term survival outcomes.

## CONCLUSION

The choice of myocardial preservation solution in HTx remains a subject of ongoing debate and research. While HTK-Custodiol offers the advantage of single-dose administration and may improve some early and mid-term outcomes like inotropic scores and rejection rates, concerns exist regarding its potential association with higher PGD rates. St Thomas and Celsior solutions demonstrate promise in reducing early post-transplant morbidity, such as the need for mechanical support, with comparable long-term survival. Overall, our data show comparable results of the 3 solutions except for severe PGD rate and temporary mechanical circulatory support. Although this data had no impact on mortality, we believe that HTK-Custodiol solution should be used with caution for preservation of donor hearts. Therefore, currently, we favour the other 2 solutions. Nevertheless, the overall impact on outcomes is multifaceted, with various studies presenting nuanced findings. Further large-scale, prospective, randomized trials are needed to definitively establish the optimal cardioplegic solution for HTx, taking into account different donor and recipient characteristics and ischaemic times.

### Limitation

The major limitation of the current investigation is the retrospective approach of the analysis. Moreover, this is a single-center study with a limited number of patients when compared to the most qualified international and national registries, and this may reduce the statistical power of some well-known risk factors. However, the completeness of data collection represents a strong point of our work.

There was no prespecified plan to adjust for multiple comparisons; hence, findings should be interpreted as exploratory in nature.

The choice of the preservation solution was left to the surgeon and this could have introduced some hidden bias and confounders in the results. Nevertheless, each surgeon showed a strong and stable preference for 1 solution during the study period.

According to the HTK-Custodiol administration protocol, a single dose of this solution was administered into the hearts of the donors unless the total ischaemia time exceeded 3 h and this, in a particular context such as cold ischaemia of HTx, could have influenced the results.

Finally, the reference group (Celsior) includes a larger number of patients and has a longer follow-up time and this may affect the results.

## Data Availability

The data underlying this article will be shared on reasonable request to the corresponding author.
